# Sensitivity and specificity of monocyte distribution width (MDW) in detecting patients with infection and sepsis in patients on sepsis pathway in the emergency department

**DOI:** 10.1007/s15010-022-01956-y

**Published:** 2022-11-18

**Authors:** Martina Cusinato, Narani Sivayoham, Timothy Planche

**Affiliations:** 1 Institute for Infection and Immunity, St. George’s, University of London, London, UK; 2 Emergency Department, St. George’s University Hospitals NHS Foundation Trust, London, UK; 3 Infection Care Group, St. George’s University Hospitals NHS Foundation Trust, London, UK

**Keywords:** Biomarker, Emergency medicine, Infections, Monocyte, Sepsis, MDW

## Abstract

**Purpose:**

Monocyte distribution width (MDW) is a biomarker for the early identification of sepsis. We assessed its accuracy in patients presenting with suspected sepsis in the emergency department (ED).

**Methods:**

This was a single gate, single centre study in consecutive adults (≥ 18 years) admitted to the ED with suspected sepsis and clinical history compatible with infection, between 01 January and 31 December 2020 (*n* = 2570).

**Results:**

The overall median MDW was 22.0 (IQR 19.3, 25.6). Using Sepsis-3 (qSOFA) to define sepsis, the Area Under Curve (AUC) for a receiver operator characteristic (ROC) relationship was 0.59 (95% CI 0.56, 0.61). Discrimination was similar using other clinical scores, and to that of C-reactive protein. At an MDW cutoff of 20.0, sensitivity was 0.76 (95% CI 0.73, 0.80) and specificity 0.35 (95% CI 0.33, 0.37) for Sepsis-3. MDW showed better performance to discriminate infection, with AUC 0.72 (95% CI 0.69, 0.75). At MDW 20.0, sensitivity for infection was 0.72 (95% CI 0.70, 0.74) and specificity 0.64 (95% CI 0.59, 0.70). A sensitivity analysis excluding coronavirus disease (COVID-19) admissions (*n* = 552) had no impact on the AUC. MDW distribution at admission was similar for bacteraemia and COVID-19.

**Conclusions:**

In this population of ED admissions with a strong clinical suspicion of sepsis, MDW had a performance to identify sepsis comparable to that of other commonly used biomarkers. In this setting, MDW could be a useful additional marker of infection.

**Supplementary Information:**

The online version contains supplementary material available at 10.1007/s15010-022-01956-y.

## Introduction

Sepsis is the primary cause of death from infection and a leading cause of critical illness, affecting millions of people worldwide annually [1]. Most cases are admitted through the emergency department (ED) following clinical deterioration of community-acquired infections [2]. Early medical intervention within hours after sepsis develops is associated with improved outcomes, so prompt and accurate identification of patients is critical [3].

The longstanding debate regarding the definition of sepsis has been attributed, in large measure, to its multifaceted nature and heterogeneous clinical phenotype. Historically, the systemic inflammatory response syndrome (SIRS) criteria have been used to define sepsis; however, although widely used, they have been criticized for their low specificity and focus on inflammation [4]. In 2016, the third international consensus definitions for sepsis and septic shock (Sepsis-3) added organ dysfunction as part of the definition to emphasise the importance of the non-homeostatic, life-threatening response to infection. At this juncture, two strategies were recommended to risk-stratify patients and clinically operationalise organ dysfunction, sequential organ failure assessment (SOFA) and quick-SOFA (qSOFA), with qSOFA recommended for settings in which all components of SOFA are not routinely measured [5]. While both scores are now used in the formal definition of sepsis, many studies have reported qSOFA to have low sensitivity and be a late indicator of deterioration [6, 7]. Further, outside intensive care unit (ICU) settings, other clinical decision tools, such as the modified early warning score (MEWS) and the national early warning score (NEWS), have been shown to better predict mortality than qSOFA while being equally easy to obtain at bedside [8–10].

Importantly, none of the risk-stratification tools available are diagnostic tests for sepsis. They identify organ dysfunction or critical illness in patients (irrespective of a certain condition) while circumventing the diagnosis of infection, which underpins the definition of sepsis. In this context, laboratory parameters and markers became an integral part of the diagnosis pathway.

Long-established markers for infection include procalcitonin (PCT) and C-reactive protein (CRP) levels. PCT has demonstrated a reasonable degree of sensitivity in identifying bacterial infections, but its high cost and variable level of evidence mean it is not often routinely used [11, 12]. In contrast, CRP is more accessible but less specific for infection and often used as a marker of the level of systemic inflammation [13, 14]. Parameters measured in the full blood count (FBC) assessment continue to be useful as first-line resources, despite their lack of specificity, and particularly since the emergence of new technologies allowing FBC to provide information on the volume and morphologic changes valuable for the diagnosis of infection and sepsis [15, 16].

The monocyte distribution width (MDW) is a novel biomarker determined in the routine FBC and has been recently CE marked as an early indicator for sepsis [17]. Recent studies have investigated the use of MDW to support a sepsis diagnosis in the ED and reported areas under the receiver operating characteristic curve (ROC AUC) that varied between 0.71 and 0.96 [18–25]. This variability possibly results from differences in study methodology and the spectrum of patients selected; but greater variability in reported specificity (range between 0.37 and 0.93) compared with sensitivity (range between 0.74 and 0.91) could also have resulted from heterogeneity within the ‘negative’ or ‘healthy’ control group across studies, i.e., the proportion of patients with non-infection cases included as controls [18–25].

This study aimed to assess the accuracy of MDW to identify sepsis in adults admitted to the ED with a high clinical suspicion of sepsis and a history suggestive of infection. The overarching goal was to challenge the marker to identify cases of sepsis in a population already screened using the National Early Warning Score 2 (NEWS2). The primary objective was formulated using the Sepsis-3 definition and qSOFA score as reference standards. As part of the exploratory objectives, this study compared MDW against other reference standards to evaluate possible changes in accuracy and facilitate its interpretation and comparability across different research paradigms.

## Methods

### Study design and participants

This was a cross-sectional, single-gate (also known as consecutive series [26]) study comparing MDW results (index test) against several clinical reference standards for sepsis (target condition). The data for this single-centre study were obtained from routinely collected medical records of patients admitted to the ED of St. George’s University Hospitals NHS Foundation Trust (SGHFT) between 01 January 2020 and 31 December 2020.

The study population was identified retrospectively by clinicians using hospital records of admissions and contemporaneous notes made by the attending clinician. Patients eligible for inclusion were consecutive adults (≥ 18 years) admitted to the ED (sepsis pathway) for suspected sepsis. The criteria for this admission route is NEWS2 ≥ 3 and a clinical history suggestive of infection or neutropenic sepsis as assessed by the ED physician on arrival [27, 28]. Exclusions were defined as patients who did not undergo an FBC assessment within 24 h of admission, had inadequate samples (e.g., error/warning messages in the results), or had been readmitted.

### Test methods

MDW results were routinely generated alongside FBC results. Following routine practice, whole human peripheral blood was collected in sterile vacutainer tubes containing K2 dipotassium ethylenediaminetetraacetic acid (EDTA-K2) and analyzed on a haematology high-volume analyser (DxH 900; Beckman Coulter, Brea, CA, USA), an established instrument for the analysis of blood samples that measures cell volume parameters and the distribution of cell volumes, as previously reported [18].

Demographic and clinical data were extracted from electronic medical records; these included vital signs, laboratory parameters at admission (including platelet count, bilirubin, creatinine, lactate and CRP levels), and clinical diagnosis of infection (based on laboratory and radiology records at admission). Data were collected by clinicians involved in patient care who were blinded to MDW values, as these were not routinely reported at the time of the study. Pathology test results (blood cultures) were retrieved from the hospital database using structured query language (SQL). Index test values and FBC results were directly downloaded from DxH 900 by a research fellow blind to the patient clinical data. All scores and target condition categorisation were calculated at the end of data collection and before merging datasets.

The primary objective defined (suspected) sepsis as participants with a documented clinical diagnosis of infection and qSOFA ≥ 2. Other definitions of sepsis studied as part of pre-defined exploratory objectives were: (i) infection and SIRS ≥ 2 (ii) infection and a change of two or more points in the baseline SOFA score (iii) infection and Risk-stratification of ED-suspected Sepsis (REDS score) ≥ 3 [29].

All scores were calculated using the component variables, as specified in the literature (see Supplementary file 1) [5, 29, 30]. For the calculation of SOFA scores, a baseline value of zero was assumed for participants not known to have pre-existing organ dysfunction, and oxygen saturation (SpO_2_) was used instead of partial pressure of oxygen (PaO_2_), as recommended in the literature [5, 30]. In addition to the scores included in Sepsis-2 (SIRS) and Sepsis-3 (qSOFA, SOFA) consensus definitions, the REDS score was used as a risk-stratification tool combining two well-known ED scores (qSOFA and simplified Mortality in Severe Sepsis in the ED score [sMISSED]) with the most commonly assessed high-risk criteria for sepsis: lactate concentration and refractory hypotension (RH) [29].

### Analysis

The distribution of baseline characteristics was assessed across the different groups (MDW ≤ 20.0 and MDW > 20.0) and analyzed using proportions, means or medians depending on the variable. The statistical tests used are stated in the table footer or figure legend, and the level of significance was 0.05.

The accuracy of MDW was assessed using standard descriptive parameters with their associated 95% confidence intervals (CIs). Exact binomial confidence limits were calculated for sensitivity, specificity, diagnostic accuracy (i.e., proportion correctly identified) and predictive values; 95% CI for positive and negative likelihood ratios were based on formulae provided by Simel et al. [31]. Hereinafter, sensitivity, specificity, overall accuracy and likelihood ratios (LRs) are discussed. Other accuracy parameters, such as predictive values and odds ratios, are included in Supplementary file 1.

The cutoff value for MDW was set at 20.0 Units as defined by the DxH 900 analyser documentation to explore dichotomous measures of accuracy [17]. The cutoff for white cell count (WCC) was set at 11 × 10^9^ cells/L, neutrophil-to-lymphocyte ratio (NLR) at 3 and 9 (corresponding to the limits of mild physiological stress [16, 32]), CRP at 20.0 mg/L and 100.0 mg/L (as an indication of mild and substantial increase [13, 14]) and lactate at 2.0 mmol/L and 3.9 mmol/L, corresponding to the component grade system used in the REDS score [29]. Because the cutoff value was used to dichotomise parameters, the exact cutoff, or a lower value ( ≤), was included in the negative category. When combining multiple parameters into one dichotomous new variable, both the conjunction (positive A and positive B) and the inclusive disjunction (positive A or positive B) were used to define positivity.

Receiver operating characteristic (ROC) curves and the corresponding area under the curve (AUC) values were calculated. Logistic regression was used to model the likelihood of the outcome as a function of MDW values in combination with other laboratory parameters. Missing data were not imputed and were handled by exclusion. Data management and statistical analysis were carried out using R (R Core Team version 3.6.3, Vienna, Austria).

The determination of sample size was guided by an expected prevalence rate of 20% for a qSOFA ≥ 2 in the study population and a point estimate of 67.9% sensitivity and 67.8% specificity for an index test cutoff value of 20.0 Units as established in the DxH 900 instructions for use [17]. The required sample size was estimated to be 1675 patients to allow for a level of precision of ± 5% in the point estimates. This target was increased by 20% (to 2010 patients) to account for the complexities of stratified analysis and missing data.

As the study was designed and approved before the start of the coronavirus disease 2019 (COVID-19) pandemic, the study population included a large and unanticipated number of patients with severe acute respiratory syndrome coronavirus 2 (SARS-CoV-2) infections admitted through the ED. All cases were confirmed by polymerase chain reaction (PCR) and/or a clinico-radiological diagnosis of COVID-19. A sensitivity analysis was conducted to explore the impact of these admissions on the study results.

### Governance and ethics

This study was approved by the Health Research Authority (20/WM/0103). Reporting follows the 2015 Standards for Reporting Diagnostic accuracy studies (STARD) guidelines statement [33]. STARD checklist is included in Supplementary file 2.

## Results

### Participants

**Fig. 1:**
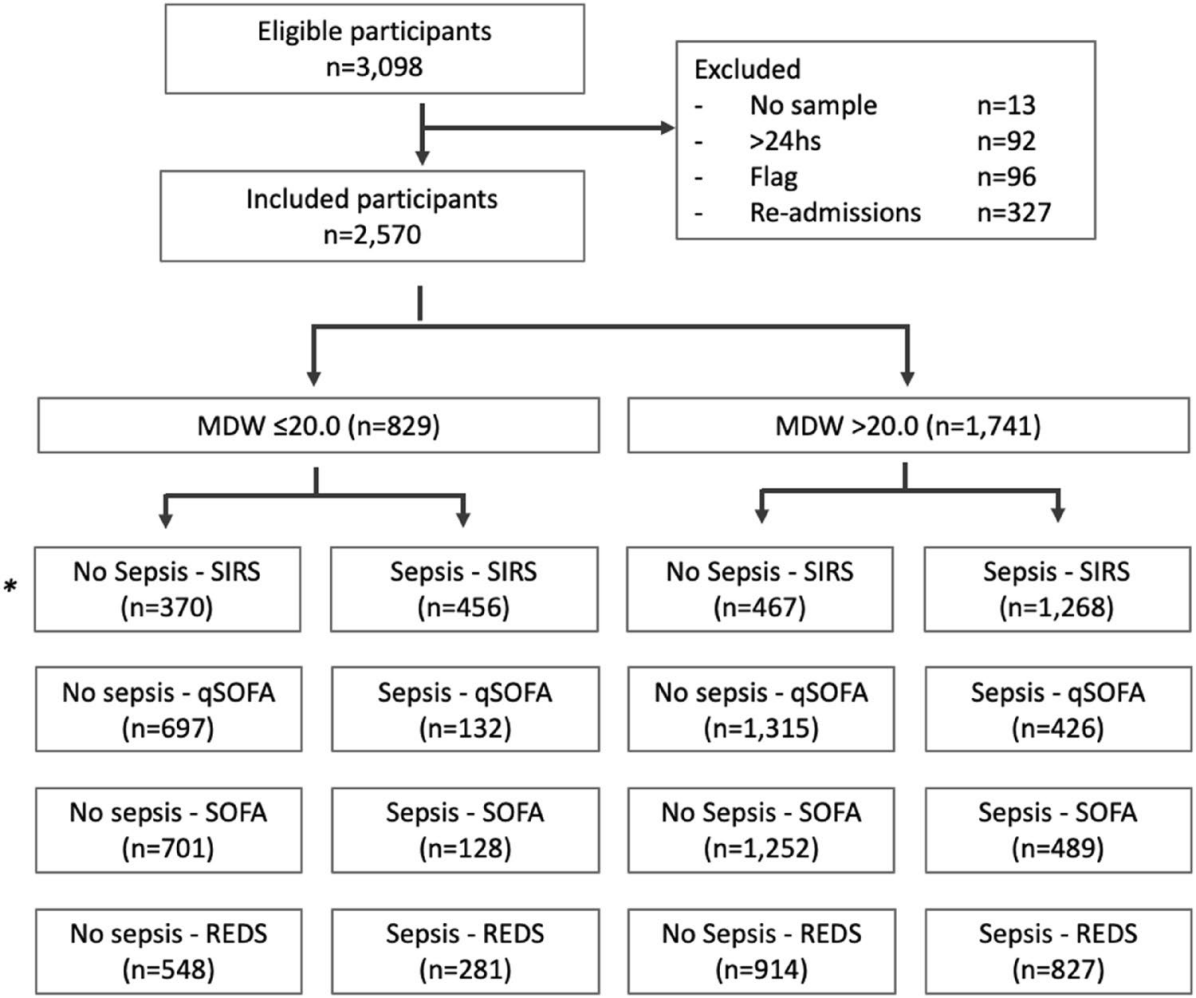
Flow of participants. Notes: * Partial verification: it was impossible to calculate SIRS in nine participants due to missing data. *>24hs* Indicates that samples were not processed within 24 hours of admission. *Flag* Indicates that the MDW parameter had a flag indicating potential issues with the MDW value requiring manual validation by technicians

Between 01 January 2020 and 31 December 2020, a total of 3098 participants met the inclusion criteria and 2570 were included in this study. Of these, 829 participants had MDW ≤ 20.0 and 1741 participants had MDW > 20. 0. Figure 1 shows the flow of patients through the study, the reasons for exclusion and the distribution of participants, with their outcomes classified using different reference standards. Verification was almost complete for all standards, with only nine participants failing to be verified for Sepsis-2 due to missing WCC values.

Patient characteristics and laboratory parameters at the time of ED admission are shown in Tables 1 and 2. In the study population, the overall prevalence of comorbid dementia and malignancy was 14.2% (365) and 12.1% (310), respectively, with weak evidence of a difference between those with MDW values over or below the cutoff value of 20.0 Units (*p* = 0.651 and *p* = 0.052). Participants with MDW ≤ 20.0 were more likely to have chronic obstructive pulmonary disease (COPD) (14.4%, 116) and to be on long-term oxygen therapy (3%, 25) than those with MDW > 20.0 (7.4%, 129 vs. 1.0%, 17 respectively). Neutropenia at admission (< 2 × 10^9^ cells/L) was not prevalent in the study population (2.5%, 64), but it was more common among those with MDW > 20.0 (3.2%, 55 of 1741).

**Table 1:** Patient characteristics at the time of ED admission

Variable		Total *n* = 2570	MDW ≤ 20.0 *n* = 829	MDW > 20.0 *n* = 1741	*p* value
Age (years)	Median (IQR)	72.0 (56.0, 83.0)	75.0 (60.0, 84.0)	71.0 (55.0, 82.0)	< 0.001
NEWS	Low	516 (20.1%)	150 (18.1%)	366 (21.0%)	< 0.001
	Low–medium	339 (13.2%)	155 (18.7%)	184 (10.6%)	
	Medium	683 (26.6%)	241 (29.1%)	442 (25.4%)	
	High	1,032 (40.2%)	283 (34.1%)	749 (43.0%)	
SIRS	0–1	661 (25.8%)	261 (31.6%)	400 (23.1%)	< 0.001
	2	887 (34.6%)	293 (35.5%)	594 (34.2%)	
	3–4	1013 (39.6%)	272 (32.9%)	741 (42.7%)	
	Missing	9	3	6	
qSOFA	0	453 (17.6%)	134 (16.2%)	319 (18.3%)	0.008
	1	1505 (58.6%)	525 (63.3%)	980 (56.3%)	
	2	559 (21.8%)	155 (18.7%)	404 (23.2%)	
	3	53 (2.1%)	15 (1.8%)	38 (2.2%)	
SOFA	Mean (SD)	2.2 (1.6)	1.9 (1.4)	2.3 (1.7)	< 0.001
	Median (IQR)	2.0 (1.0, 3.0)	1.0 (1.0, 2.0)	2.0 (1.0, 3.0)	
	Range	1.0–13.0	1.0–10.0	1.0–13.0	
REDS	0–2	1,332 (51.8%)	472 (56.9%)	860 (49.4%)	< 0.001
	3–4	905 (35.2%)	285 (34.4%)	620 (35.6%)	
	5–6	248 (9.6%)	61 (7.4%)	187 (10.7%)	
	≥ 7	85 (3.3%)	11 (1.3%)	74 (4.3%)	
Infection	No	297 (11.6%)	191 (23.0%)	106 (6.1%)	< 0.001
	Yes	2273 (88.4%)	638 (77.0%)	1635 (93.9%)	
Septic	No	2526 (98.3%)	825 (99.5%)	1701 (97.7%)	< 0.001
Shock	Yes	44 (1.7%)	4 (0.5%)	40 (2.3%)	
Blood	Negative	2406 (93.6%)	809 (97.6%)	1597 (91.7%)	< 0.001
Culture	Positive	164 (6.4%)	20 (2.4%)	144 (8.3%)	
COVID-19	Negative	2018 (78.5%)	784 (94.6%)	1234 (70.9%)	< 0.001
	Positive	552 (21.5%)	45 (5.4%)	507 (29.1%)	

The table shows column proportions. *p* values correspond to the chi-squared test for categorical variables and Kruskal–Wallis for continuous variables

**Table 2:** Laboratory parameters at the time of ED admission

Variable		Total *n* = 2570	MDW ≤ 20.0 *n* = 829	MDW > 20.0 *n* = 1741	*p* value
WCC	Median (IQR)	11.1 (7.7, 15.3)	11.6 (8.4, 14.9)	10.9 (7.1, 15.5)	0.009
	Missing	9	3	6	
Neutrophil	Median (IQR)	8.9 (5.7, 12.8)	9.2 (6.2, 12.2)	8.8 (5.4, 13.1)	0.346
count	Missing	9	3	6	
Lymphocyte	Median (IQR)	1.0 (0.6, 1.5)	1.2 (0.7, 1.7)	0.9 (0.6, 1.4)	< 0.001
count	Missing	9	3	6	
NLR	Median (IQR)	8.8 (5.0, 16.2)	7.8 (4.3, 13.4)	9.3 (5.3, 17.4)	< 0.001
	Missing	11	3	8	
CRP	Median (IQR)	78.0 (29.0, 159.0)	31.0 (8.4, 80.5)	105.0 (48.0, 191.2)	< 0.001
Level	Missing	19	10	9	
Lactate	Median (IQR)	1.5 (1.0, 2.3)	1.6 (1.0, 2.4)	1.5 (1.0, 2.3)	0.679
level	Missing	0	0	0	

The table shows cell counts in 10^9^ cells/L, CRP levels in mg/L, lactate in mmol/L. *p* values correspond to Kruskal–Wallis test. NLR was missing for two participants with lymphocyte counts of zero

**Fig. 2:**
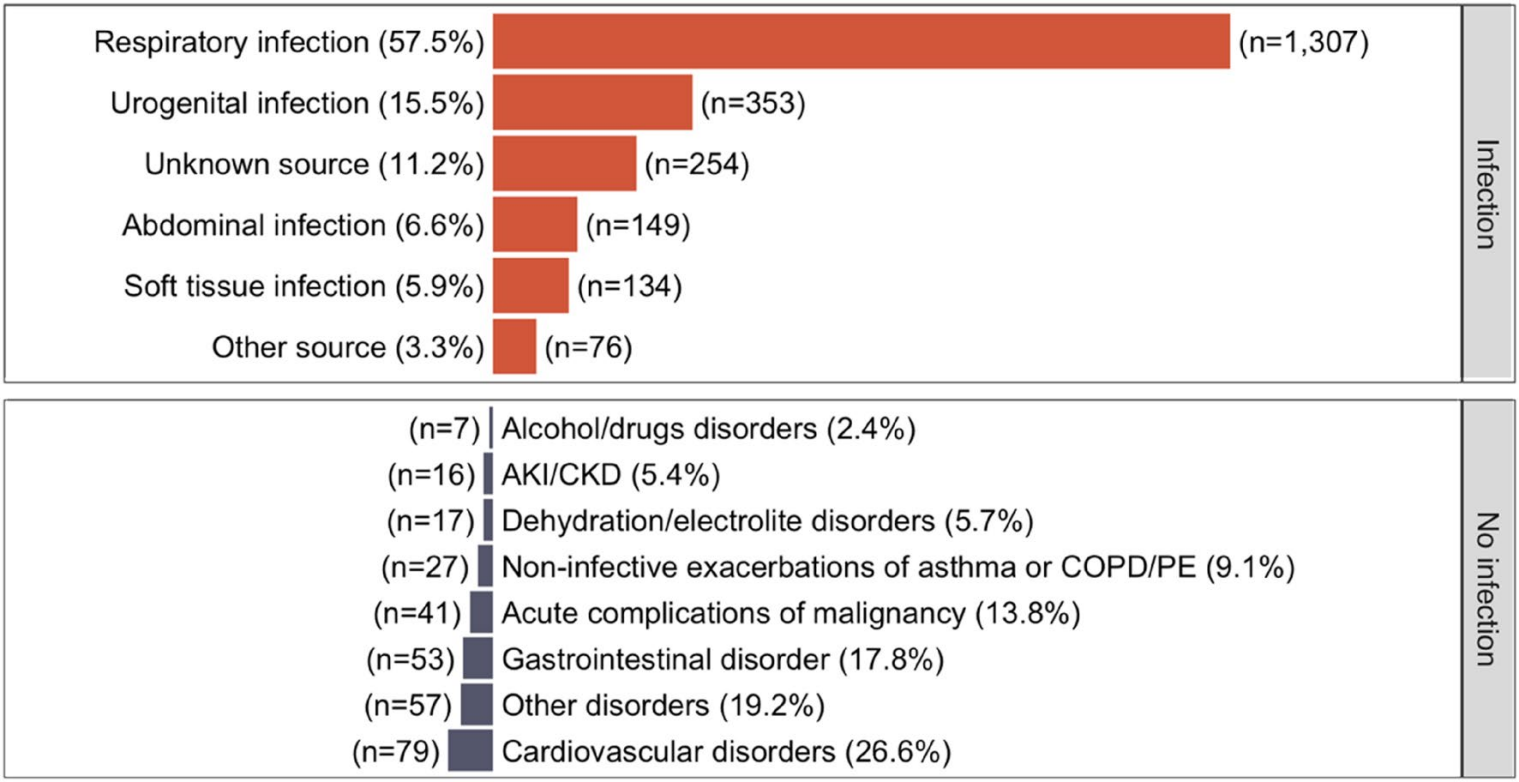
Distribution of main diagnoses at admission. The plot shows proportions per strata (infection and no infection) and absolute frequency (n). *Other source* Other known sources of infection. *AKI* Acute kidney injury. *CKD* Chronic kidney disease. *Other disorders* included seizures, rash, diabetes ketoacidosis, hyperosmolar hyperglycaemic syndrome (HHS), headache, vomiting, urinary retention

Sepsis suspicion at ED admission and a clinical history suggestive of infection were integral components of the inclusion criteria in this study. Consequently, the population had a large prevalence of patients with infection diagnoses (2273, 88.4%). The distribution of infection sources is displayed in Fig. 2 together with the main reason for admission in those eventually found not to have an infection (297, 11.6%). The distribution of scores at admission (Table 1) shows the overall study population had a moderate to high risk of deterioration. Positive blood cultures (excluding common commensal microorganisms [34]) were more prevalent among those with MDW > 20.0 (8.3%, 144) and COVID-19–positive admissions were also more prevalent in this group (29.1%, 507).

### Test results

**Fig. 3:**
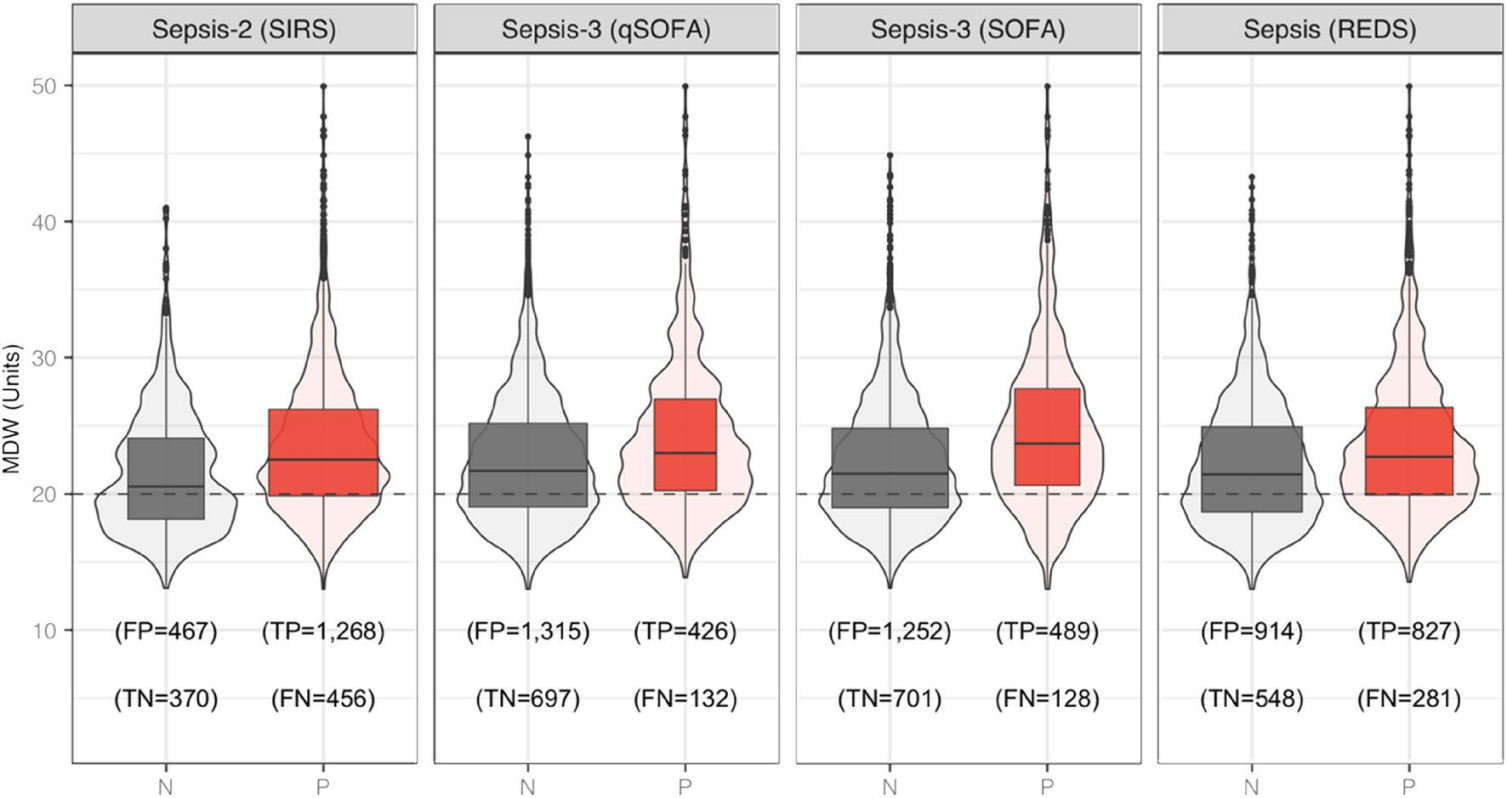
MDW distribution across sepsis for different sepsis definitions. *N* Negative for sepsis; *P* Positive for sepsis; *FP* False positive; *TP* True positive; *FN* False negative; *TN* True negative. The width of the box plots is proportional to the size of the category

The mean MDW value in the overall study population was 23.0 (standard deviation [SD] 5.4), the median 22.0 (IQR 19.3, 25.6) and the range 13.0–85.0. The distribution of MDW values according to different reference standards for sepsis is shown in Fig. 3. Regardless of the reference standard used, approximately 75% of those classified as sepsis had MDW values greater than 19.9. However, the distribution of MDW values overlapped considerably among positive and negative sepsis groups (across all reference standards). The degree of overlap was smaller when the reference was Sepsis-2 (infection and SIRS ≥ 2), which means there was a smaller proportion of false (positive and negative) classifications.

Point estimates of diagnostic accuracy (and 95% CI) for MDW across reference standards are shown in Table 3. As illustrated in Fig. 3, the sensitivity was relatively high, but the specificity was low. Using MDW 20.0 as the cutoff, the point estimate for sensitivity remained between 0.74 and 0.79 across all references, but the specificity dropped well below 0.50. The highest diagnostic accuracy was observed using MDW in concert with Sepsis-2 at the expense of improving specificity, which yielded the largest positive LR. Depending on the reference, point estimates for AUCs ranged from 0.58 to 0.63. Table 3 shows that excluding COVID-19 patients (sensitivity analysis) had no impact on the AUC.

**Table 3:** Estimates of MDW diagnostic accuracy across different definitions of sepsis

	Sepsis-2 SIRS	Sepsis-3 qSOFA	Sepsis-3 SOFA	Sepsis REDS
Sensitivity	0.74	0.76	0.79	0.75
(95% CI)	(0.71, 0.76)	(0.73, 0.80)	(0.76, 0.82)	(0.72, 0.77)
Specificity	0.44	0.35	0.36	0.37
(95% CI)	(0.41, 0.48)	(0.33, 0.37)	(0.34, 0.38)	(0.35, 0.40)
Accuracy	0.64	0.44	0.46	0.54
(95% CI)	(0.62, 0.66)	(0.42, 0.46)	(0.44, 0.48)	(0.52, 0.55)
LR Positive	1.32	1.17	1.24	1.19
(95% CI)	(1.23, 1.41)	(1.10, 1.24)	(1.17, 1.30)	(1.13, 1.26)
LR Negative	0.60	0.68	0.58	0.68
(95% CI)	(0.54, 0.67)	(0.58, 0.80)	(0.49, 0.68)	(0.60, 0.76)
AUC^a^—*n* = 2570	0.62	0.59	0.63	0.58
(95% CI)	(0.59, 0.64)	(0.56, 0.61)	(0.60, 0.65)	(0.56, 0.61)
AUC^b^—*n* = 2018	0.63	0.59	0.64	0.59
(95% CI)	(0.61, 0.66)	(0.56, 0.62)	(0.61, 0.67)	(0.57, 0.62)

The table shows sensitivity, specificity and diagnostic accuracy as proportions and calculated for a cutoff MDW ≤ 20.0. AUC stands for the area under the curve

^a^Refers to full sample size, *n* = 2570 except for SIRS when *n* = 2061

^b^Refers to subpopulation removing COVID-19 positive patients, *n* = 2570 except for SIRS when n = *2*014

### Additional parameters

**Fig. 4:**
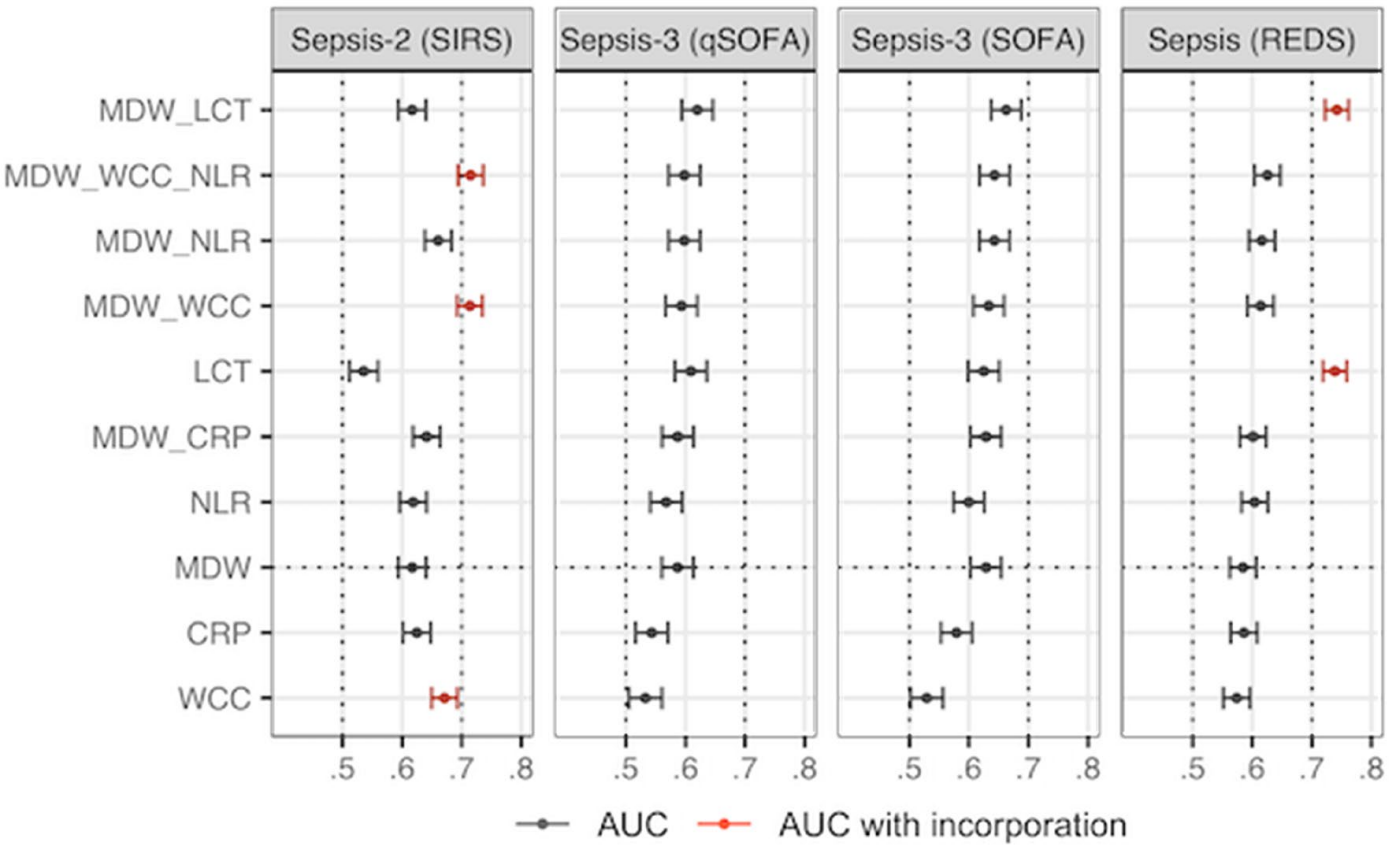
Comparison of AUC for MDW and other sepsis markers. *LCT* stands for lactate levels. *ACUs* were calculated on complete cases. There were 19 participants with missing CRP, two with missing NLR and nine with missing WCC and NLR

The diagnostic accuracy of MDW was compared against other parameters used in ED: WCC and NLR, which are both determined via FBC analysis alongside MDW, as well as the CRP level and lactate concentration measured at admission. The AUC (and 95% CI) values for these parameters, isolated and in combination with MDW, are shown in Fig. 4. AUC values were relatively consistent across the different reference standards, with magnitudes ranging between 0.50 and 0.70. The AUC values for WCC and lactate concentration were probably overestimated for Sepsis-2 and Sepsis using the REDS score, as the definition of these scores incorporates WCC and lactate. These AUCs should be interpreted with caution (shown in red in Fig. 4). AUCs 95% CI for MDW and CRP showed a high degree of overlap when using SIRS and REDS scores, but when using Sepsis-3 (SOFA or qSOFA) instead, the overlap was reduced, with MDW observing higher AUC values. As expected, assessment of the lactate concentration yielded a significantly lower AUC in concert with Sepsis-2.

**Fig. 5:**
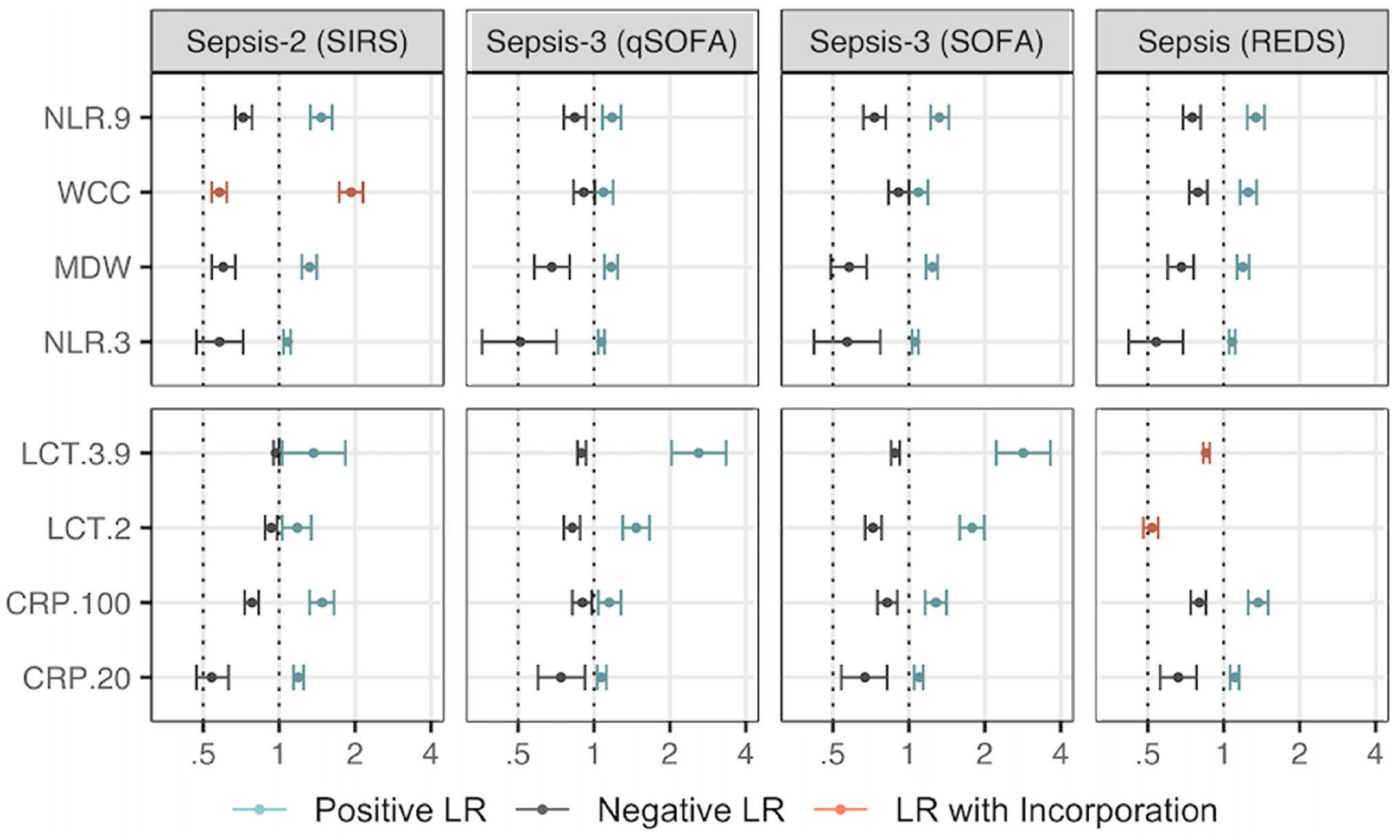
Positive and Negative LR for MDW, WCC, NLR, CRP and lactate. The x-axis is shown in log-scale. *LCT* stands for lactate levels. The numerical values following the marker indicate the cutoff value, as detailed in the methods section. Cutoff values are not printed when the marker has only one cutoff value (i.e. MDW cutoff value is 20.0 and WCC is 11 x 109 cells/L). The positive LRs using REDS score for LCT3.9 (6.60) and LCT2 (4.30) are not shown to facilitate visualisation of lower values in the LR scale. There were 19 participants with missing CRP, 11 with missing NLR and nine with missing WCC

**Fig. 6:**
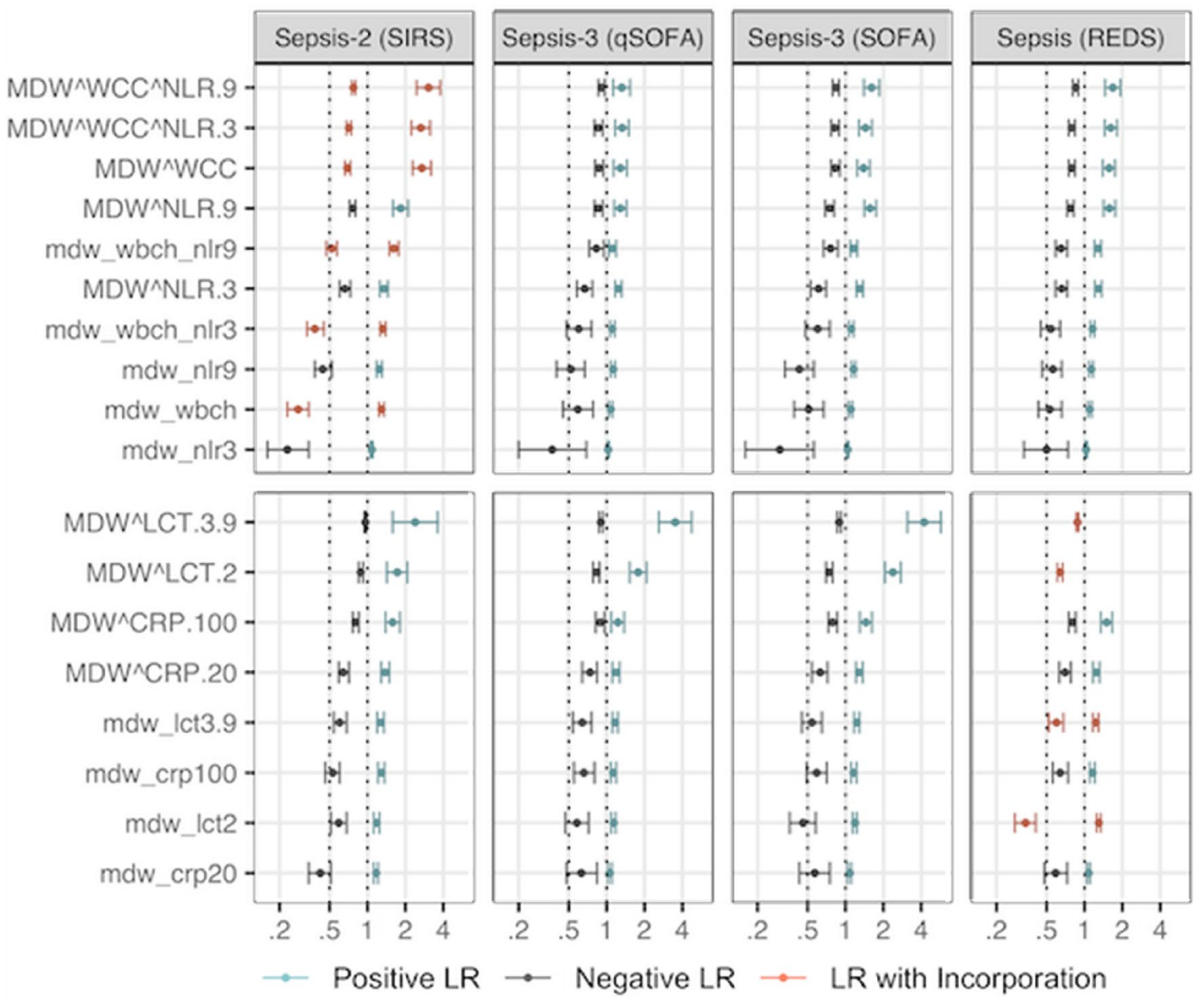
Positive and Negative LR for MDW combined with other FBC parameters, CRP and lactate. The x-axis is shown in log-scale. Parameters in *capital letters* and connected using ‘Ù’ indicate conjunction ('and'). Parameters in *lowercase letters* and connected using ‘_’ indicate disjunction (or). *LCT* stands for lactate levels. The numerical values following the marker indicate the cutoff value, as detailed in the methods section. Cutoff values are not printed when the marker has only one cutoff value (i.e. MDW cutoff value is 20.0 and WCC is 11x109 cells/L). The positive LRs using REDS score for MDW_LCT.3.9 (14.31) and MDW.LCT2 (6.40) are not shown to facilitate visualisation of lower values in the LR scale. There were 19 participants with missing CRP, 11 with missing NLR and nine with missing WCC

As a next step, we explored the dichotomisation of parameters to estimate the probability of the outcome for a given test result and focused on LRs because these can be adapted to varying prior probabilities of the disease, unlike predictive values (shown in supplementary materials), which are rarely generalisable beyond the study sample [35]. The trade-off between positive and negative LRs was explored for different parameters alone (Fig. 5) and in combination (Fig. 6), using the cutoff values specified under methods. The top panel shows FBC parameters (MDW, WCC and NLR), and the bottom panel shows lactate levels and CRP values in both figures. Estimates for WCC and lactate need to be interpreted with caution in concert with SIRS and REDS scores due to incorporation.

Figure 5 shows that all single parameters LRs remained significant, although in some cases the strength of the evidence was weak. MDW > 20.0 was between 17 and 32% more likely in sepsis patients than in non-septic patients, depending on the reference score used (positive LR 1.17 and 1.32) and MDW ≤ 20.0 was between 32 and 42% more likely in non-septic patients than in septic patients (negative LR 0.58 and 0.68). The largest positive LR (point estimate) unbiased by incorporation was observed for lactate > 3.9 mmol/L using Sepsis-3 as reference (2.84 when using SOFA, and 2.60 with qSOFA), and the smallest negative LRs were observed for NLR > 3 (between 0.51 and 0.58 depending on the reference), MDW > 20.0 (between 0.58 and 0.68) and CRP > 20.0 mg/L (between 0.54 and 0.74). Notably, the magnitude of the point estimates for negative LRs did not fall below 0.5, so a negative result was, at most, 50% more likely in a non-sepsis case (true negative) than in a sepsis case (false negative).

Figure 6 displays LRs for MDW combined with other parameters, using both conjunction (‘and’) and disjunction (‘or’). The former emphasises the identification of true positive cases and the latter the identification of true negative cases. The combination yielding a maximum improvement in positive LRs (unbiased by incorporation) was observed for ‘MDW > 20.0 and lactate > 3.9 mmol/L’. In this combination and using Sepsis-3 as reference, a positive value was 3.51 (qSOFA) or 4.22 (SOFA) times more likely in sepsis compared to non-sepsis). The maximum improvement in negative LRs was seen for the combination ‘MDW > 20.0 or NLR > 3’ (inclusive disjunction declares the positive test), so ‘MDW ≤ 20.0 and NLR ≤ 3’ (note the reversal of the inequality symbol) were 70% (SOFA) and 63% (qSOFA) more likely in a true negative sepsis case than in a false negative sepsis case (negative LR 0.30 and 0.37 respectively). ‘MDW ≤ 20.0 and CRP ≤ 20.0 mg/L’, as well as ‘MDW ≤ 20.0 and NLR ≤ 9’ observed negative LR and 95% CI fully below 0.5, but only for Sepsis-2.

### Infection, inflammation and organ dysfunction

**Fig. 7:**
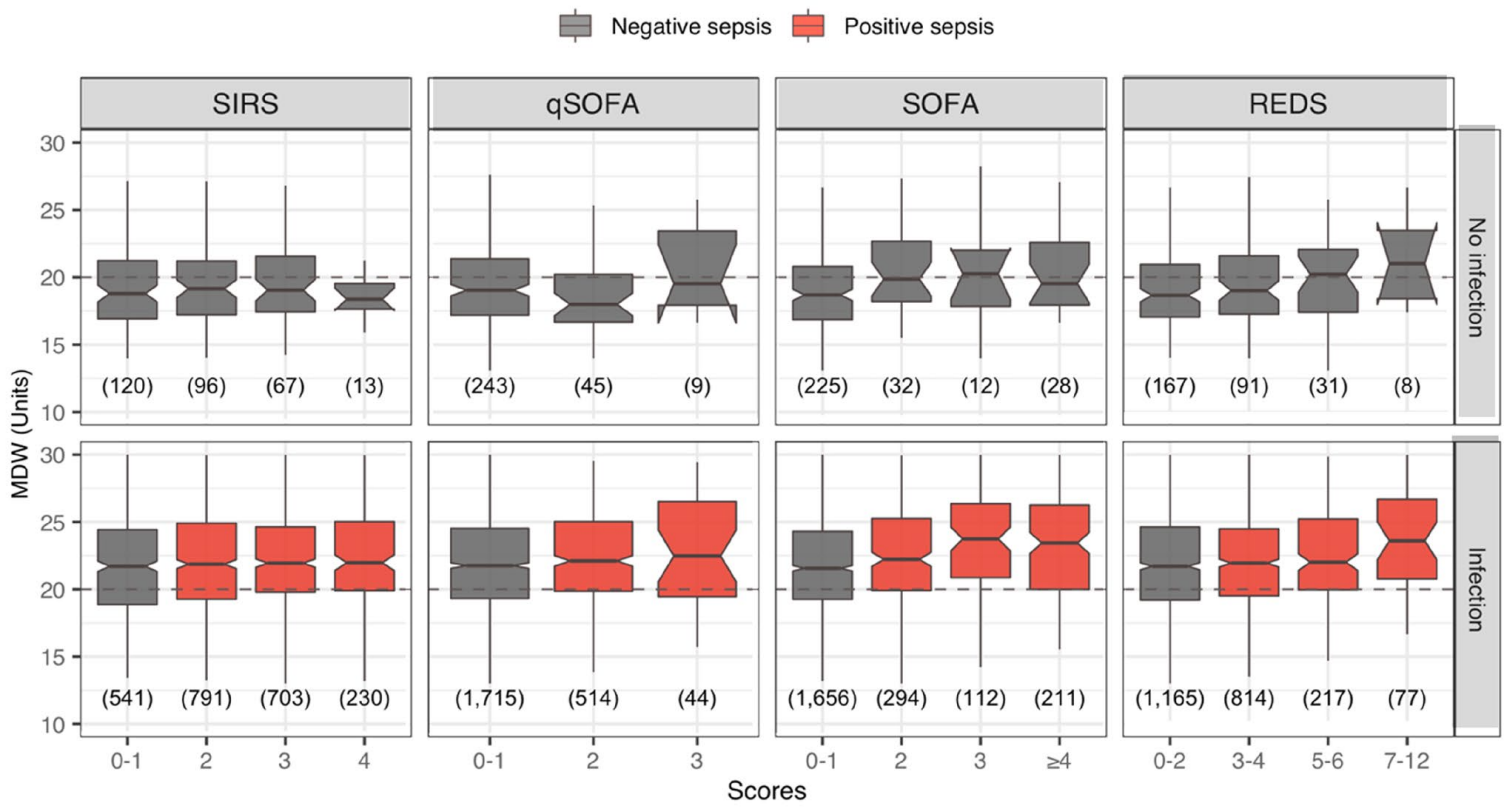
MDW distribution across sepsis scores and according to presence or absence of infection diagnosis. The number of observations is included in parentheses (n). The *box plots* exclude outliers (y-axis ranges from 10 to 30 Units) as the focus is on showing the centre of the MDW distributions relative to the cutoff value. The width of the box plots is not proportional to the size of the category to aid visualisation, although the size of each group is indicated below each box. The 95% CI for the median is shown as notches/indentations around the median value. Inverted notches indicate that the median 95% CI exceeds the corresponding quartile

Figure 7 shows the distribution of MDW values across the different sepsis scores, stratified according to the diagnosis of infection. MDW distributions in the non-infection stratum (top row) were predominantly below the MDW cutoff value in contrast with the infection stratum (bottom row), in which roughly 75% of the MDW distributions (from first quartile up) were above 20.0. There were some statistically significant increments in the median MDW values for the higher categories of REDS and SOFA scores (illustrated in Fig. 7 by non-overlapping notches or median 95% CI). However, these differences were minor in magnitude and should be interpreted in the context of overlapping box plot distributions. Figure 7 also illustrates that SIRS ≥ 2 classified 76.1% (1,724 of 2,265) of infections as sepsis, whereas qSOFA and SOFA scores identified less than 27.1%, and the REDS score 48.7% (1108 of 2273).

An ad hoc calculation of MDW accuracy to identify infection rather than sepsis produced, in this population, an AUC value of 0.72 (95% CI 0.69, 0.75); and at MDW ≤ 20.0 a sensitivity of 0.72 (95% CI 0.70, 0.74), specificity of 0.64 (95% CI 0.59, 0.70), positive LR of 2.02 (95% CI 1.73, 2.35) and negative LR of 0.44 (95% CI 0.39, 0.49).

**Fig. 8:**
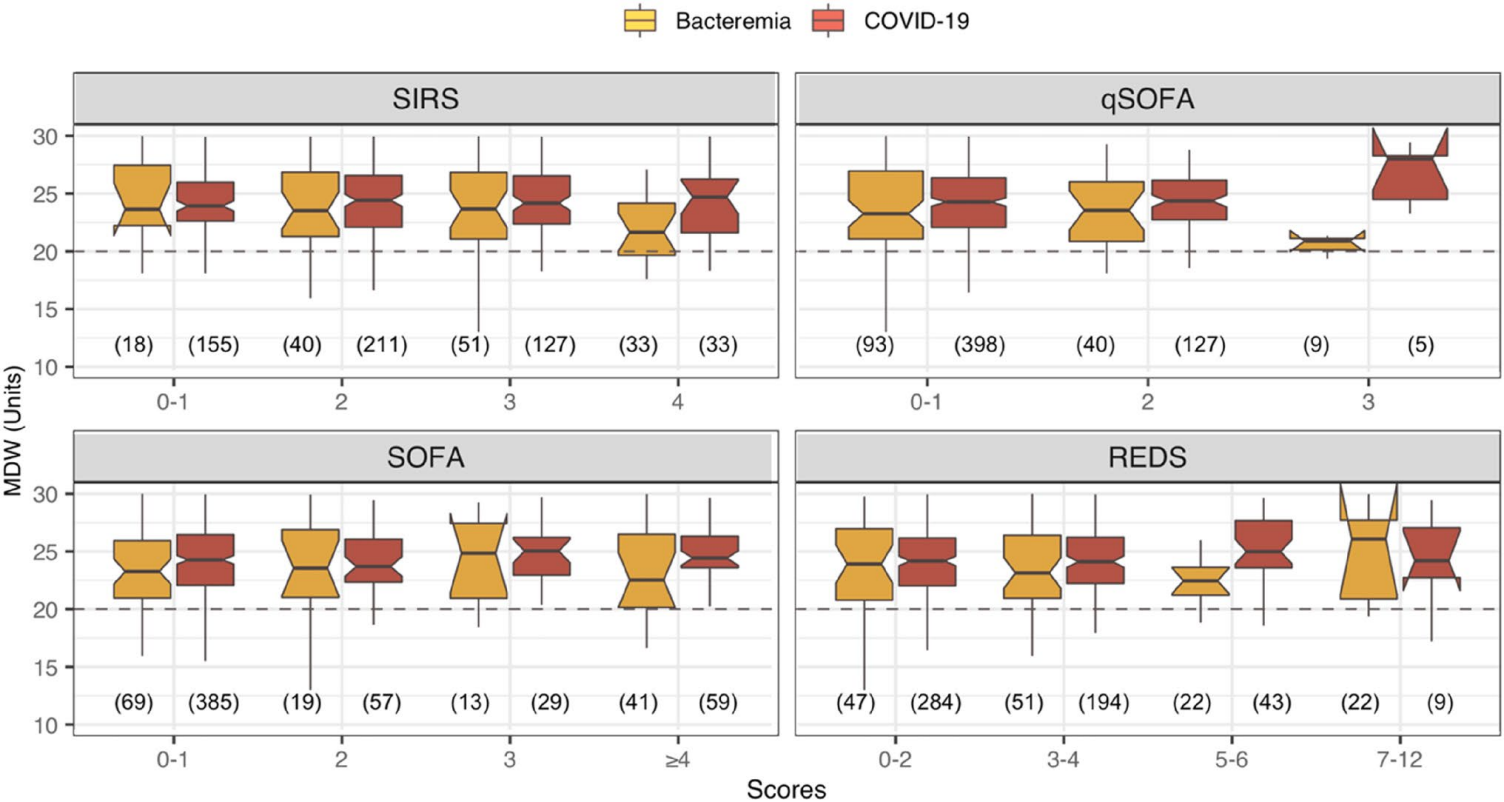
MDW distribution across sepsis scores in patients with either bacterial or COVID-19 infection. The number of observations is included in parentheses (n). The *box plots* exclude outliers (y-axis ranges from 10 to 30 Units) as the focus is on showing the centre of the MDW distributions relative to the cutoff value. The width of the box plots is not proportional to the size of the category to aid visualisation, although the size of each group is indicated below each box. The 95% CI for the median is shown as notches/indentations around the median value. Inverted notches indicate that the median 95% CI exceeds the corresponding quartile

Figure 8 shows MDW values across different sepsis scores for a subset of infections, i.e., those with confirmation of bacteraemia and COVID-19 infection. In both subsets, 50% of MDW values around the median (IQR) remained above 20.0, consistent with Fig. 7. COVID-19 MDW distributions had narrower IQRs and slightly higher median values than bacterial infections, although COVID-19 was a larger group. Figure 8 shows the absence of a clinically meaningful trend of MDW increase according to any sepsis score (for each infection type).

The prevalence of septic shock (defined as RH and maximum lactate > 2 mmol/L [5]) was 1.7% (44 of 2570). The median MDW among septic shock patients was 29.3 (IQR 22.8, 37.0). All cases of septic shock had SOFA scores ≥ 2 and REDS scores ≥ 5. RH with maximum lactate ≤ 2 mmol/L was observed in 1.0% of the population (25 of 2570), showing median MDW values of 23.9 (IQR 21.4, 26.9), as well as SOFA and REDS scores ≥ 3.

## Discussion

This study assessed the accuracy of MDW to identify sepsis in a large population of patients requiring admission to the ED with a strong clinical suspicion of sepsis (*n* = 2570). This population had an overall distribution of MDW values (median 22.0, IQR 19.3, 25.6) higher than of other (unselected) ED admissions during the same study period who had an FBC assessment requested but were not on the sepsis pathway (*n* = 53,850, median 17.3, IQR 15.8, 18.8). The selection criteria strongly challenged the marker in classifying the target condition.

The MDW AUC values observed in this study were relatively consistent across the different reference standards, with point estimates ranging between 0.58 and 0.63. Other parameters assessed at admission (WCC, NLR, CRP and lactate concentration) generated comparable AUC values, and the overlapping 95% CI (Fig. 4) shows that the overall performance to identify sepsis (independently of the reference standard) was relatively similar for all parameters when considering incorporation. Combining MDW with other laboratory parameters only marginally improved the performance level for identifying sepsis. These findings are consistent with studies that included fewer participants without infection in their sample [22, 23].

In this population**,** using MDW 20.0 as cutoff, sensitivity remained between 0.74 and 0.79, with positive LRs between 1.17 and 1.32 across all references. However, the point estimates for specificity dropped below 0.44, rendering point estimates for negative LRs between 0.68 and 0.58. So, MDW ≤ 20.0 was between 32 and 42% more likely in a non-sepsis case than in a sepsis case. Although effect sizes were small, these results indicate that MDW ≤ 20.0 (on its own) might perform slightly better at identifying true negative cases when the population includes a low proportion of non-infection cases. Combining MDW with lactate resulted in large increments of positive LR (for Sepsis-3) compared to either parameter alone. This study observed no differences in the distribution of MDW values between confirmed bacteraemia and COVID-19 infections across comparable score levels (Fig. 8).

This study assessed several clinical reference standards, minimising partial verification bias, and observed comparable levels of accuracy across the different definitions of sepsis, with a slight increase in specificity when using Sepsis-2, concordant with other research [36]. Given that SIRS is more accurate in diagnosing inflammation than organ dysfunction, and qSOFA/SOFA identify cases of organ dysfunction more precisely, the observed increased diagnostic accuracy (of MDW ≤ 20.0) for Sepsis-2 may have been due to the infection component in the definition of sepsis rather than better discrimination of sepsis. Consistent with this observation, MDW appeared to better identify infection (AUC 0.72, 95% CI 0.69, 0.75) than sepsis (ad hoc analysis). Although this study was not powered to test this hypothesis, this might have been why accuracy was lower when using Sepsis-3 definitions (the accuracy was underestimated because the index test and reference standard were measuring different things).

This study was carried out over an entire calendar year (2020), encompassing all four seasons, but concurrently with the COVID-19 pandemic. Although the sensitivity analysis did not find differences in the performance of MDW to identify sepsis when including or excluding COVID-19 cases, the study population might not be representative of non-pandemic times and could be biased towards severity. Misclassification is a frequent limitation of observational studies. Even though systematic misclassification was addressed by blinding both the MDW results and the target condition, random misclassification cannot be ruled out. Downregulation of the immune system has been found to increase MDW values in septic patients [22], but participants were not excluded based on their haematology disorder or immunocompetent status since this study sought to closely reflect real-world practice. There was good compliance with manufacturer instructions regarding the time elapsed from venepuncture to FBC analysis; the median time was 0.1 h (IQR 0.1, 0.6) with only two samples exceeding the recommended two hours. There was no statistically significant difference in the distribution of time from venepuncture to analysis (*p* = 0.322) or missing values in this variable (*p* = 0.829) across study groups. Overall, there was a good level of data completeness.

This study examined a population of patients with suspected sepsis admitted to the ED of a reference teaching hospital in London, England and its results may only be generalised to the adult ED population routinely screened using NEWS2 or a comparable score system.

## Conclusion

This study analyzed a population of patients requiring admission to the ED with a high clinical suspicion of sepsis. In this population (with a small proportion of non-infection cases), MDW performance to identify sepsis was similar to that of other commonly used biomarkers. An increment of MDW values of small magnitude was observed along with higher REDS scores; larger increments were noted in a small subgroup of admissions with septic shock. In this population, MDW distribution at admission was similar for bacteraemia and COVID-19. Results suggest MDW could be an effective marker of infection in routine clinical use.

## Supplementary Information

Below is the link to the electronic supplementary material.

Supplementary file1 (XLSX 474 KB)

Supplementary file2 (DOCX 34 KB)

## Data Availability

The data underlying this article are available in the article and in its online supplementary material.

## Declarations

### Patient and public involvement

The patients and the public were involved in the development of the study protocol. Patient organisations might be further involved in disseminating these data to inform local management and policy.
